# BaGdF_5_ Nanophosphors Doped with Different Concentrations of Eu^3+^ for Application in X-ray Photodynamic Therapy

**DOI:** 10.3390/ijms222313040

**Published:** 2021-12-02

**Authors:** Zaira Gadzhimagomedova, Vladimir Polyakov, Ilia Pankin, Vera Butova, Daria Kirsanova, Mikhail Soldatov, Darya Khodakova, Anna Goncharova, Elizaveta Mukhanova, Anna Belanova, Aleksey Maksimov, Alexander Soldatov

**Affiliations:** 1 The Smart Materials Research Institute, Southern Federal University, 344090 Rostov-on-Don, Russia; vlpolyakov@sfedu.ru (V.P.); pankin@sfedu.ru (I.P.); vbutova@sfedu.ru (V.B.); dkirsanova@sfedu.ru (D.K.); mikhailsoldatov@sfedu.ru (M.S.); kand@sfedu.ru (E.M.); soldatov@sfedu.ru (A.S.); 2 National Medical Research Centre for Oncology, 344037 Rostov-on-Don, Russia; hodakovadv@rnioi.ru (D.K.); goncharovaas@rnioi.ru (A.G.); maksimovay@rnioi.ru (A.M.); 3 Faculty of Chemistry, Southern Federal University, 344090 Rostov-on-Don, Russia; 4 Academy of Biology and Biotechnologies, Southern Federal University, 344090 Rostov-on-Don, Russia; abelanova@sfedu.ru

**Keywords:** X-ray photodynamic therapy, nanophosphor, photosensitizer, reactive oxygen species, tumor, computed tomography, scintillating nanoparticles

## Abstract

X-ray photodynamic therapy (XPDT) has been recently considered as an efficient alternative to conventional radiotherapy of malignant tissues. Nanocomposites for XPDT typically consist of two components—a nanophosphor which re-emits X-rays into visible light that in turn is absorbed by the second component, a photosensitizer, for further generation of reactive oxygen species. In this study, BaGdF_5_ nanophosphors doped with different Eu:Gd ratios in the range from 0.01 to 0.50 were synthesized by the microwave route. According to transmission electron microscopy (TEM), the average size of nanophosphors was ~12 nm. Furthermore, different coatings with amorphous SiO_2_ and citrates were systematically studied. Micro-CT imaging demonstrated superior X-ray attenuation and sufficient contrast in the liver and the spleen after intravenous injection of citric acid-coated nanoparticles. In case of the SiO_2_ surface, post-treatment core–shell morphology was verified via TEM and the possibility of tunable shell size was reported. Nitrogen adsorption/desorption analysis revealed mesoporous SiO_2_ formation characterized by the slit-shaped type of pores that should be accessible for methylene blue photosensitizer molecules. It was shown that SiO_2_ coating subsequently facilitates methylene blue conjugation and results in the formation of the BaGdF_5_: 10% Eu^3+^@SiO_2_@MB nanocomposite as a promising candidate for application in XPDT.

## 1. Introduction

X-ray photodynamic therapy (XPDT) is a relatively new approach to treating cancer diseases [1]. The basic principle of XPDT is similar to that of the traditional photodynamic therapy (PDT) [2,3,4,5]. In PDT, nanocomposites are introduced into abnormal cells and irradiated by ultraviolet–visible light (UV–vis). However, UV–vis radiation has low tissue penetration. Thus, X-rays may be a promising source for deep photodynamic activation due to overcoming the penetration capability [6,7,8]. In XPDT, nanocomposites consist of nanophosphors and photosensitizers (PSs). Injected nanocomposites are irradiated with X-ray beams and then nanophosphors reradiate X-rays into UV–vis fluorescence. The converting energy is used in the activation process of photosensitizers for further generation of reactive oxygen species (ROS) that destroy the vascular system of malignant tissues.

**Figure 1:**
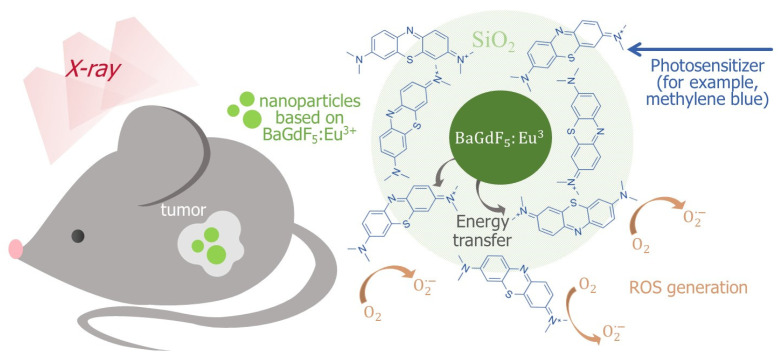
Schematic illustration of the BaGdF_5_:Eu^3+^-assisted XPDT concept and possible morphology of the corresponding composite material based on the BaGdF_5_:Eu^3+^ agent coupled with the methylene blue photosensitizer.

Nanophosphors based on BaGdF_5_ are of particular interest to the application in X-ray photodynamic therapy [9,10,11,12,13,14,15]. They efficiently absorb ionizing radiation in the X-ray range and readily convert it into visible light [9,10,14,16,17,18,19,20] (Figure 1). Many works have been conducted to obtain such Ln^3+^-doped BaGdF_5_ nanomaterials. For example, BaGdF_5_ nanoparticles (NPs) doped with Yb^3+^, Ho^3+^ have been synthesized with different morphologies, paramagnetic properties, higher down-conversion and up-conversion luminescence via a facile additive assisted hydrothermal route [21]. Another hydrothermal method was used in [22] to obtain BaGdF_5_:Eu^3+^ phosphors. The most intensive band on the luminescent spectra was observed at 592 nm. In another investigation, Yang et al. synthesized BaGdF_5_:Yb^3+^/Er^3+^ nanoparticles via active shell modification, revealing that enhancement of the up-conversion luminescence of BaGdF_5_:Yb^3+^/Er^3+^ nanoparticles and that luminescence colors of the active-core/active-shell nanoparticles can be tuned from green to yellow by means of multilayer active-shell coating [23].

BaGdF_5_:Ce^3+^, Eu^3+^, BaGdF_5_:Ce^3+^, Tb^3+^ were synthesized in [18] using the hydrothermal method. Transmission electron microscope images revealed an average size of 12 nm. All the samples demonstrated luminescence under ultraviolet irradiation and emitted rays in the visible range. The investigated nanoparticles exhibited a paramagnetic property. Cytotoxicity studies against human erythrocytes indicated that the synthesized nanoparticles were nontoxic because they did not cause red blood cell shape, membrane structure and permeabilization changes.

BaGd_1–x–y–z_F_5_: xYb^3+^, yEr^3+^, zEu^3+^ dual-mode luminescence nanophosphors were synthesized in [12] using a simple one-step hydrothermal method. Under 274 nm UV light excitation, nanophosphors emitted orange light. Under 980 nm near-infrared radiation, nanophosphors intensively emitted visible green light. The enhanced up-conversion luminescence of the BaGdF_5_: Yb^3+^, Er^3+^/Eu^3+^ nanophosphors was realized by modifying the trisodium citrate surfactant. The trisodium citrate surfactant-modified and unmodified nanoparticles all had sphere-like morphology, with an average size of 25 and 44 nm, respectively. Furthermore, the nanoparticles had a paramagnetic feature in previously discussed investigations [18,21].

Monodispersed BaLnF_5_:Yb^3+^(20%)/Er^3+^(2%) (Ln = La^3+^, Gd^3+^, Lu^3+^) nanocrystals were synthesized in [24] using a thermal decomposition method. According to the investigation, the size of the nanocrystals decreased with the Ln^3+^ ionic radius in the BaLnF_5_ structure without affecting the phase and shape of the nanocrystals. The luminescence efficiency also followed the same trend as the size of the nanoparticles.

BaGdF_5_:Yb^3+^/Tm^3+^ conjugated with anti-cancer drug doxorubicin nanoparticles was reported in [10]. According to the transmission electron microscopy imaging, the size of nanoparticles was approximately 10 nm. Doxorubicin can be selectively released by the cleavage of hydrazone bonds in an acidic environment, which shows a pH-triggered drug release behavior. Long-term in vivo toxicity studies indicated that mice intravenously injected with 10 mg/kg of the nanoparticles survived for 40 days without any apparent adverse effects on their health.

Indeed, nanophosphors based on BaGdF_5_ have low cytotoxicity [25]. Zhigao Yi et al. synthesized BaGdF_5_: x% Ln^3+^ (x = 0, 50 or 100; Ln^3+^ = Dy^3+^, Er^3+^ or Yb^3+^) nanoparticles, which demonstrate high luminescence and paramagnetic properties [25]. The viability of Hela cells for these nanoparticles was above 79.5% with 500 μg/mL concentrations. In vitro cell viability studies revealed the low cytotoxicity of the nanoparticles. In vitro hemolytic assay showed excellent blood compatibility of the nanostructures. The authors reported that BaGdF_5_: 50% Er^3+^ nanophosphors can emerge as synergistic contrast-enhancing agents for tumor detection in T_1_/T_2_ dual-weighted MRI.

In addition, biological macromolecules are larger than nanoparticles. The size of nanoparticles is usually in the order of tens of nanometers, which is 1000 times smaller than the size of a cancer cell. That is, unlike microparticles, nanoparticles show good cellular uptake. This makes them ideal candidates for the delivery of anticancer drugs [26]. Because of their small size, nanomaterials can overcome biological barriers. Small capillaries have a diameter of about 3 micrometers, and nanoparticles with a size of 200 nm can be freely transported through the circulatory system and carry pharmaceutically active substances.

Effectiveness of the drugs used in biomedicine also strongly depends on their biocompatibility with target cells. It is well-known that the outer surface of the lipid bilayer of the cell membrane is negatively charged. Therefore, endocytosis depends on the nanoparticle surface charge [27]. In addition, neutral nanoparticles have a lower endocytosis rate compared to charged nanoparticles, while positive and negative nanoparticles have different clearance speed and biodistribution [28,29]. In turn, neutral and negatively charged nanoparticles have a long circulation period [28]. Due to endocytosis, the efficiency of internalization is highly dependent on the nanoparticles charge. Clathrin-mediated endocytosis allows positively charged particles to be selectively internalized [27]. However, in spite of the higher speed of endocytosis of positively charged nanoparticles, they accumulate on the vascular endothelium without subsequent penetration into tissues or tumors, unlike their neutral and negatively charged counterparts [28]. Furthermore, positively charged nanoparticles evade lysosomes, causing disruption in their functioning, leading to swelling and rupture [30]. Therefore, for biomedical applications, the most optimal is a neutral or negative charge of the surface of nanoparticles.

To obtain particles with a negatively charged surface, nanoparticles are often coated with surface ligands. Citric acid or amino acids (glutamic or aspartic) are usually used as biocompatible ligands [31,32,33,34]. This choice is due to the presence of two or more carboxyl groups. One of the carboxyl groups forms a chelate complex with Ln^3+^ ions while the remaining groups create a negative surface charge, which promotes dispersion of the particles due to electrostatic repulsion. It was also found that the stability of colloidal particles coated with a citrate depends on pH. With pH decrease from 7.0 to 4.5, a stable colloid begins to precipitate particles. However, when an alkali is added to the system, the precipitate is dispersed. These pH-dependent coagulation/dispersion processes are reversible many times over [35].

In this work, X-ray-scintillating nanoparticles BaGdF_5_:Eu^3+^ with varied Eu:Gd ratio and controlled size distribution were obtained through microwave synthesis. We systematically considered the surface post-treatment with PEG and citrates for enhanced biocompatibility and amorphous SiO_2_ which showed notably enhanced methylene blue (MB) photosensitizer conjugation for the construction of BaGdF_5_:Eu^3+^@SiO_2_@MB nanocomposites. In addition, synthesized NPs demonstrate efficient X-ray attenuation, thus making the obtained nanocomposites a promising candidate for both XPDT and CT imaging.

## 2. Results

### 2.1. BaGdF_5_:Eu^3+^ Nanophosphors

#### 2.1.1. Crystal Structure and Composition

The elemental composition of the BaGdF_5_ samples doped with the following different initial concentrations (x = 1, 2.5, 5, 10, 25, 50%) of Eu^3+^ was assessed using X-ray fluorescence analysis (XRF). The actual elemental composition percentage and molar ratio of the Ba, Gd and F elements for each sample were calculated and reported in Supplementary Table S1, while the expected and actual percentage of Eu^3+^ was reported in Table 1. The obtained results demonstrate that the Eu^3+^ precursor almost entirely reacts during the synthesis involved in NP formation and that BaGd_1–x_F_5_Eu_x_ stoichiometry is well-established. Some notable deviations between the predicted and actual Eu content observed for the lowest Eu loading were likely due to the presence of a tiny amount of the nonreacted Eu precursor as well as an XRF experimental error in determination at the low-concentration level.

**Figure 2:**
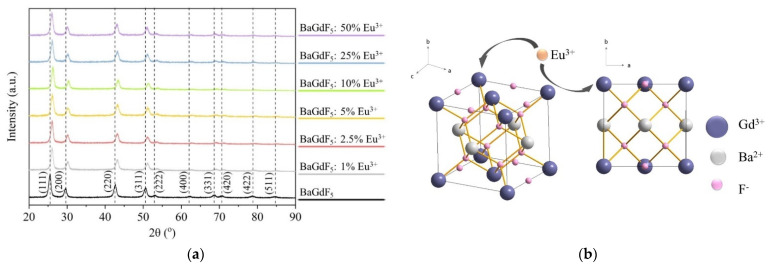
(**a**) X-ray diffraction patterns of the synthesized samples BaGdF_5_ and BaGdF_5_: x% Eu^3+^ (x = 1, 2.5, 5, 10, 25, 50); (**b**) schematic illustration of the BaGdF_5_ unit cell with Eu^3+^-doped sites.

X-ray powder diffraction (XRD) profiles were collected for the BaGdF_5_ nanoparticles doped with different amounts of Eu^3+^ reported in Figure 2a. Qualitative examination revealed single-phase material formation for all the BaGdF_5_: x% Eu^3+^ samples with a structure similar to those reported for undoped BaGdF_5_ in PDF database JCPDS No. 24-0098, space group Fm-3m and cell parameter a = 6.023 Å, V = 218.49 Å (Figure 2b). No detectable traces of a side Eu-containing product or amorphous phase were observed, thus demonstrating that the overwhelming majority of Eu^3+^ ions were incorporated into the BaGdF_5_ matrix.

Cell parameters were calculated from profile analysis using the Jana2006 software program package (Table 1). The value of the BaGdF_5_: x% Eu^3+^ cell parameters were close to the value of the non-doped BaGdF_5_ sample a = 5.909 Å, but Eu doping led to the tiny modification of cell parameters. This can be clearly observed by the gradual shift of diffraction maxima with Eu content increase. The obtained cell parameter is smaller than the PDF database value (a = 6.023 Å). This phenomenon of crystal lattice compression is associated with the size effect of nanoparticles [36]. In addition, the crystalline size was estimated using the Scherrer equation and the Williamson–Hall (W–H) analysis (see Table 1). 

**Figure 3:**
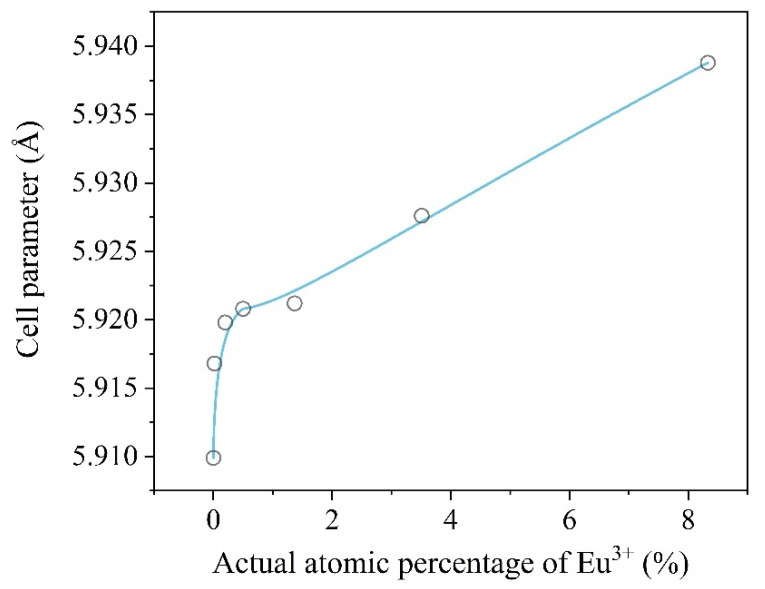
Correlation between the actual percentage of doping Eu^3+^ ions and the refined lattice parameter.

In Figure 3, one can see that cell parameters increased with the increase in the Eu^3+^ doping percentage. This can be explained by the fact that Eu^3+^ R_i_ = 1.087 Å [37] has a larger ionic radius compared to Gd^3+^ R_i_ = 1.078 Å [37]. The obtained trend and particularly its linear character at higher Eu concentrations confirms that Eu^3+^ ion is inserted into the lattice structure of BaGdF_5_. Thus, cell parameters depend on the amounts of doping elements.

The purity of the final products was monitored by means of Fourier-transform infrared spectroscopy (FTIR) (see Figure S1) and discussed in the Supplementary Materials.

The amount of PEG can be estimated by means of thermogravimetric analysis (TGA). Comparison of the TGA and differential scanning calorimetry (DSC) curves performed on PEG-modified and pure BaGdF_5_: 10% Eu^3+^ is reported in Figure S2. A slight difference in the weight loss in the range of 150–350 °C apparently can be associated with the elimination of PEG molecules from the surface.

#### 2.1.2. Transmission Electron Microscopy Analysis and Particle Size Distribution

**Figure 4:**
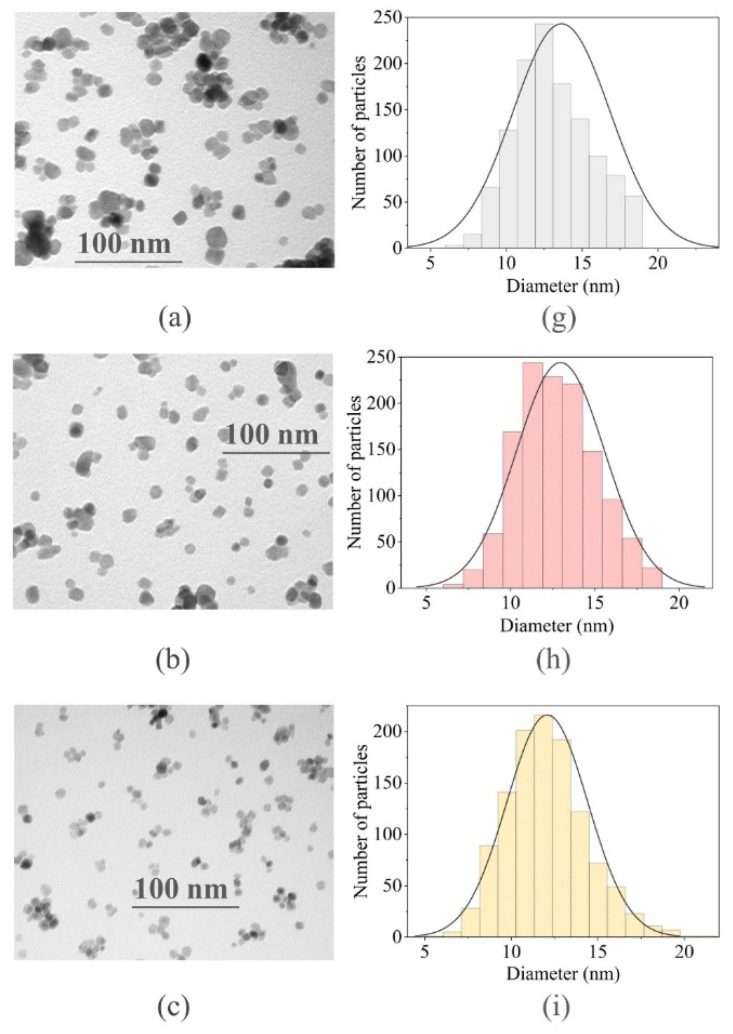

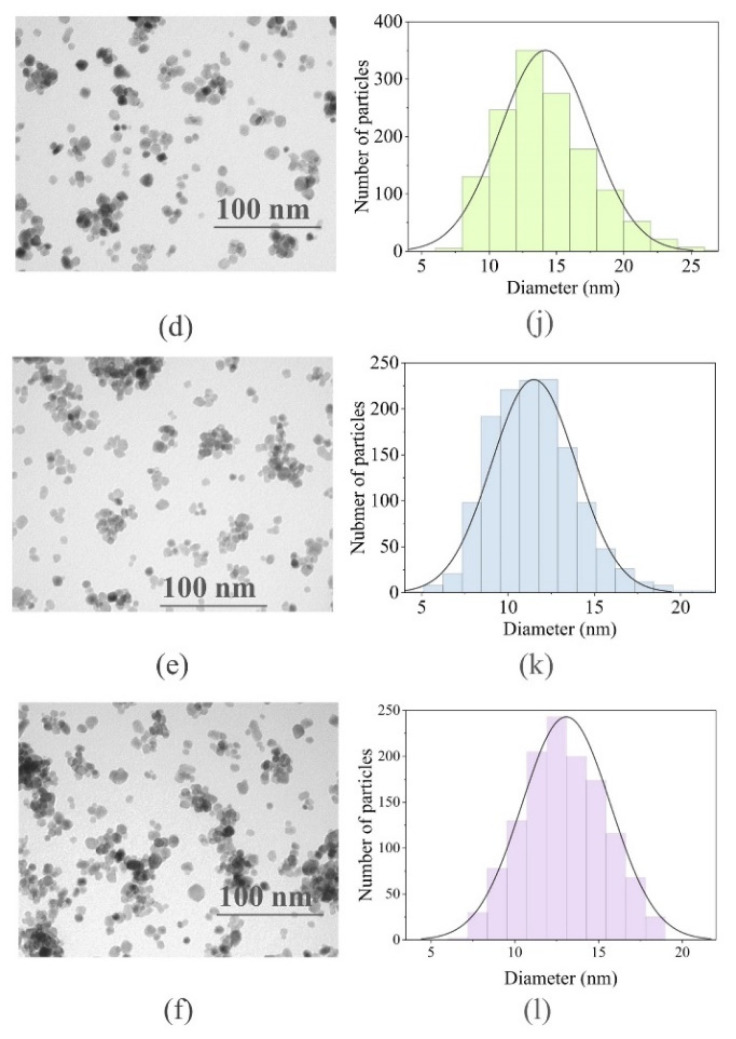
(**a**–**f**) TEM images of the synthesized BaGdF_5_: x% Eu^3+^ samples, where x = 1, 2.5, 5, 10, 25, 50%, respectively; (**g**–**l**) particle size distribution of BaGdF_5_: x% Eu^3+^ obtained from TEM image analysis.

The shape, morphology and size of the nanoparticles were studied using transmission electron microscopy (TEM). As seen in TEM images and from the analysis reported in Figure 4, all the BaGdF_5_: x% Eu^3+^ samples were spherical particles.

The size distribution of the nanoparticles was estimated using the ImageJ program. The total amount of nanoparticles taken into account for analysis was 1300 for the 1% and 2.5% doped samples, 1160 for the 5% doped sample, 1374 for the 10% doped sample, 1360 for the 25% doped sample and 1300 for the 50% doped sample. It was found that the nanoparticle size distribution peaked at approximately 11 nm for the 2.5% and 5% doped samples, at 12 nm for the 1%, 25% and 50% doped samples and at 13 nm for the 2.5% doped sample.

The sizes (small) of the synthesized samples were appropriate for further coating with SiO_2_. Indeed, such small nanoparticles are known to readily overcome biological barriers [26]. Small capillaries have a diameter of about 3 micrometers, and nanoparticles with a size less than 200 nm can be freely transported through the circulatory system and carry pharmaceutically active substances.

#### 2.1.3. X-ray-Excited Optical Luminescence

Rare-earth metal ions such as Eu^3+^ doped into a wide range of materials exhibit optical luminescence upon UV–vis and X-ray excitation [13,20,38,39]. The latter makes it possible to convert ionizing radiation into visible light as part of the XPDT system. On the other hand, a study of certain transition intensities in X-ray-excited optical luminescence (XEOL) spectra could give additional information about the environment of the lanthanide ion.

**Figure 5:**
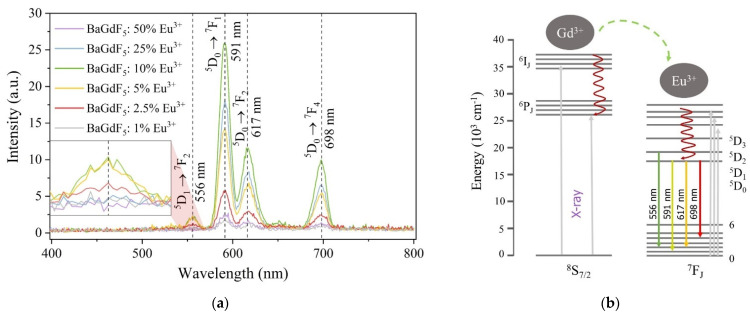
(**a**) X-ray-excited (U = 35 kV, I = 1.6 mA) optical luminescence spectra measured for BaGdF_5_ doped with x% of Eu^3+^; (**b**) proposed scheme of the energetic process occurring in BaGdF_5_:Eu^3+^.

The XEOL spectra reported in Figure 5a for a series of BaGdF_5_: x% Eu^3+^ samples show typical spectral shape for the Eu^3+^-doped BaGdF_5_ matrix. The observed luminescence spectrum of the synthesized NPs resulted from transition within the f-electron of the Eu^3+^ ions. The observed peaks in the optical range were related to the Eu^3+^ forbidden electric-dipole and magnetic-dipole 4f → 4f (^5^D_0_ → ^7^F_J = 0,1,2,3,4_) transitions (as shown in Figure 5b) which strongly depend on the symmetry of the Eu^3+^ sites [39,40,41]. While magnetic-dipole ^5^D_0_ → ^7^F_1_ (λ = 591 nm) and ^5^D_0_ → ^7^F_4_ (λ = 698 nm) transitions are relatively insensitive to the environment, low intensity of electric-dipole transition ^5^D_0_ → ^7^F_2_ (λ = 617 nm) as well as the absence of the ^5^D_0_ → ^7^F_0_ transition could be a sign of high symmetry of the Eu^3+^ site in the synthesized materials.

It should also be emphasized that the presence of a weaker band ^5^D_1_ → ^7^F_2_ (λ = 556 nm) was observed only for the sample with a moderate content of Eu, i.e., 2.5%, 5% and 10% of Eu, see inset in Figure 5a. The low-phonon energy of the fluoride matrix caused a weak probability of quenching due to multiphonon relaxation, which explains the observation of such radiation at short wavelengths [42]. The peak intensity increased with an increase in doping material Eu^3+^ from 2.5% to 10% and then decreased with an increase of the Eu concentration up to 50%. The peak intensity was not in direct correlation with the number of radiation centers. Probably, it is related to the effect of concentration like previously observed quenching in some Eu^3+^-doped samples [42].

**Figure 6:**
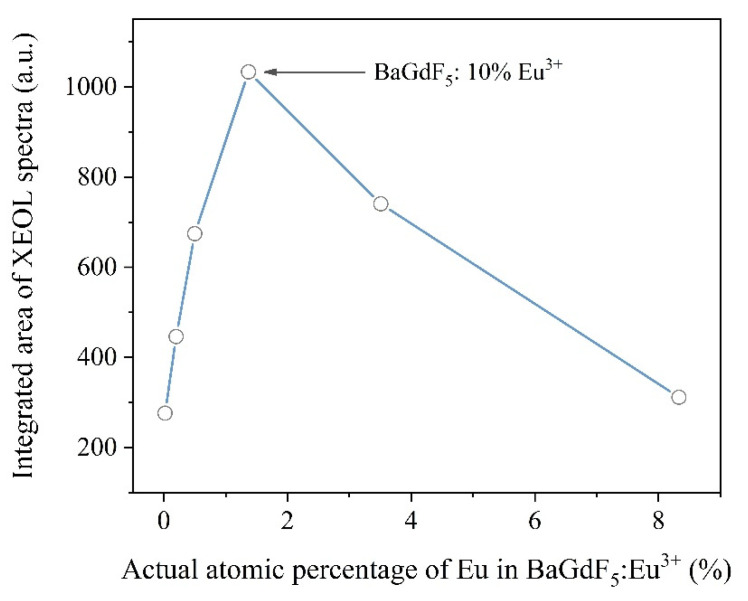
Correlation between the integrated area of XEOL spectra (400–800 nm range) and the actual atomic percentage of Eu in the BaGdF_5_: x% Eu^3+^ samples.

It is worth mentioning that except for the low-intensity peak centered at λ = 556 nm, the relative intensity between other peaks remained the same. Thus, the total X-ray-excited luminescence efficiency of nanophosphors can be estimated as signal intensity integrated over the range from 400 to 800 nm (as reported in Figure 6). Finally, we may declare that the optimal actual content of doped Eu is equal to 1.37 at. % (i.e., BaGdF_5_: 10% Eu^3+^).

#### 2.1.4. Nanoparticles’ Colloidal Solution Stability

Dynamic light scattering (DLS) measurements directly characterize particles in dispersion and allow monitoring of dispersion effects such as the time effect on colloidal stability. Moreover, hydrodynamic sizes of nanoparticles can be estimated from DLS data. Thus, during the study, the stability of the solution and the hydrodynamic size of the dissolved nanoparticles were verified. All the samples were dissolved in 10 mL of distilled water with a concentration of 2 mg/mL.

**Figure 7:**
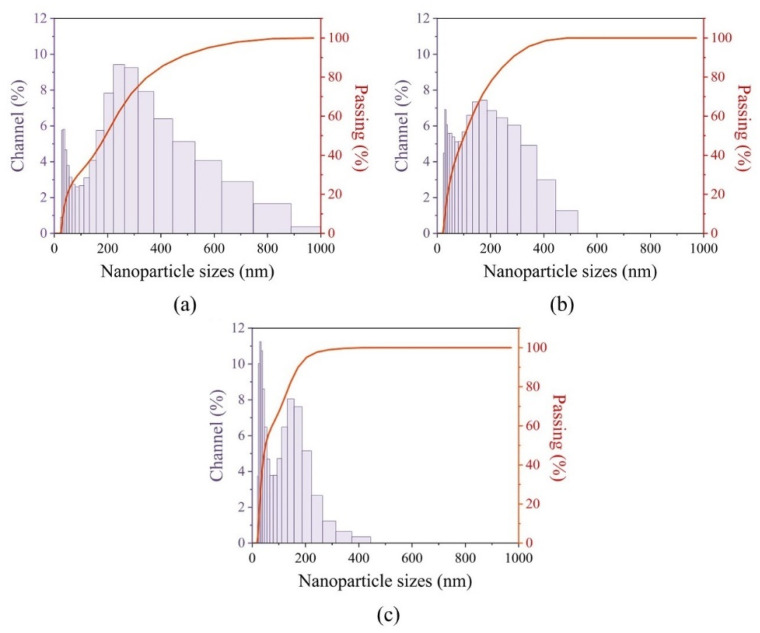
Dynamic light scattering measurements of the BaGdF_5_: 10% Eu^3+^ sample for 1 (**a**), 5 (**b**) and 30 (**c**) minutes after stirring processes.

In case of the BaGdF_5_: 10% Eu^3+^ sample after 1 min of data acquisition, a predominantly large fraction of the sample in the colloidal solution was observed (Figure 7a). After 5 min, the amount of the large fraction decreased (Figure 7b). Mostly smaller nanoparticles with a hydrodynamic radius of about 30 nm remained in the colloidal solution. The large fraction was likely to form a precipitate (Figure 7c).

Thus, the solution with the BaGdF_5_: 10% Eu^3+^ nanoparticles was stable for half an hour. Moreover, a large fraction of the sample fell into the precipitate, leaving the nanoparticles of smaller sizes in the colloid.

As can be seen from DLS data (Figure 7c), hydrodynamic sizes of nanoparticles in the established colloid (after 30 min measurements) ranged from 30 nm to 400 nm. The large fraction was due to the presence of aggregated nanoparticles in the solution (Figure 7a). It is worth noting that the hydrodynamic size of the nanoparticles is always greater than their real size. Therefore, the hydrodynamic size estimated from DLS was substantially different from the size distribution obtained via TEM microscopy (Figure 4) and X-ray diffraction calculations (Table 1).

Z-potential is the electrical potential in the slip plane. The plane separates the moving fluid from the fluid that is attached to the surface. Ζ-potential is usually used as a detector of droplet stability. Zeta-potential values above +30 mV or below −30 mV indicate good stability of aqueous dispersions [43]. Thus, the stability of the solution can be verified via ζ-potential measurements. 

Ζ-potential of the BaGdF_5_ and BaGdF_5_: x% Eu^3+^ samples is reported in Table 2. All the samples were dissolved in 2 mL of distilled water with a concentration of 2 mg/mL. No clear trends were observed in the measured ζ-potentials with the variation of the Eu amount in the samples. The average ζ-potential of all the samples was +29.95 mV, revealing that the BaGdF_5_ and the BaGdF_5_: x% Eu^3+^ suspensions were stable in an aqueous solution.

### 2.2. Citrate Surface Modification

**Figure 8:**
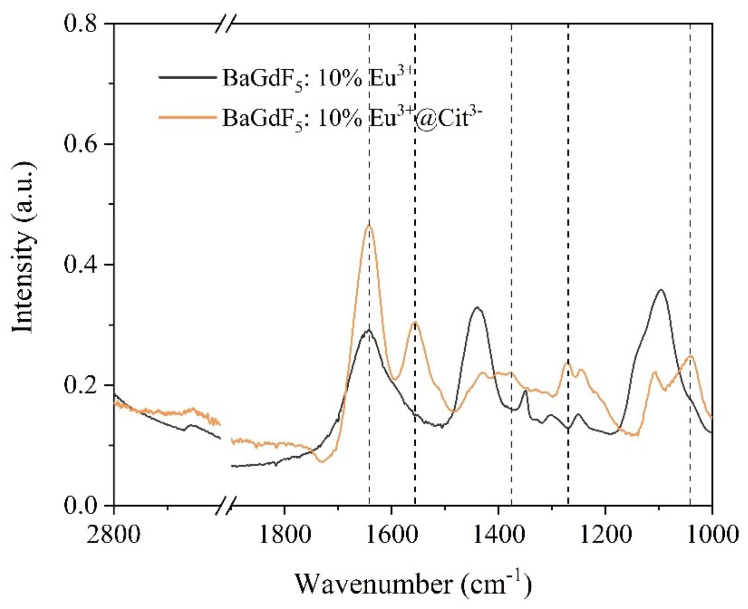
FTIR spectra of BaGdF_5_: 10% Eu^3+^ and BaGdF_5_: 10% Eu^3+^@Cit^3−^.

For surface modification with a citrate and any further nanoparticle manipulations, the BaGdF_5_: 10% Eu^3+^ sample was selected, as it turned out to be the most promising in terms of X-ray scintillating efficiency. To confirm the particle surface modification, FTIR spectra of the unmodified and citrate-coated nanoparticles were acquired (Figure 8). The spectra of the coated sample displayed characteristic acyl C=O bands at 1560 and 1378 cm^−1^ attributed to the asymmetrical and symmetrical stretching modes, respectively [44]. Furthermore, we observed bands at 1041 and 1271 cm^−1^ corresponding to citrate C−H bending and acyl C−O asymmetrical stretching, respectively [45]. The increase in the peak at 1643 cm^−1^ was associated with an increase in the water molecules’ amount adsorbed on the particles’ surface. These bonds were in agreement with the analogous modification of Fe_3_O_4_ magnetite nanoparticles [46].

To assess stability of the modified particles in the colloidal solution form, measurements of their ζ-potential were carried out at pH = 7. As can be seen, the sign of the ζ-potential changed from positive to negative. At the same time, the absolute value of the ζ-potential remained quite high (−30 mV). The alternating surface charge of the coated nanoparticles is strong evidence that the coating of the oppositely charged components was successful. Nanoparticle dispersions with ζ-potential values of ± 20–30 mV are classified as moderately stable in the drug delivery literature [47]. The obtained results demonstrated that nanoparticles’ surface modification with citrates facilitates better stability of colloidal solutions, which is important for the possible application in CT imaging. Thus, at the next stage, we explored an X-ray attenuation capability and possible application as a contrast agent for Cit^3−^-coated BaGdF_5_: 10% Eu^3+^ nanoparticles.

#### CT Imaging and Biodistribution

Prior to the in vivo CT imaging we estimated the X-ray attenuation capability of the synthesized materials by comparison with commercial iodine-based nonionic contrast agent Optiray-350^®^. For these purposes, we chose citrate-coated BaGdF_5_: 10% Eu^3+^ due to the known positive effect of citrate coating on the biocompatibility of nanoparticles [48,49,50]. An aqueous solution of nanoparticles and the commercial Optiray contrast agent were prepared with different concentrations of I and heavy elements in the nanoparticle core, namely Ba and Gd (44.5 mM, 31.1 mM, 18 mM), and X-ray attenuation efficiency was verified.

**Figure 9:**
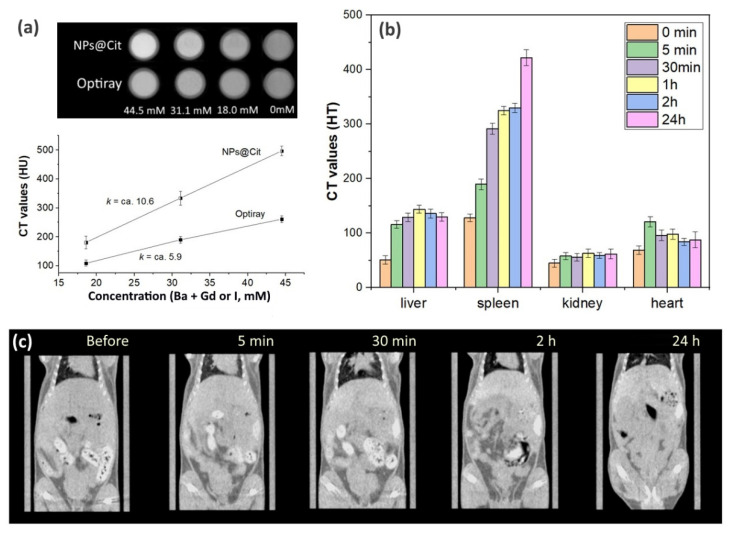
(**a**) In vitro CT imaging for the aqueous solution of the citrate-coated BaGdF_5_: 10% Eu^3+^ nanoparticles and commercial iodine-based contrast agent Optiray-350. (**b**) Time evolution of HU values registered for different mouse organs after an intravenous injection of the citrate-coated BaGdF_5_: 10% Eu^3+^ nanoparticles (HU values before the injection reported for the sake of comparison) and (**c**) the corresponding CT images taken before and after the injection.

Similarly to the previously reported results for BaGdF_5_-based systems [10,51,52,53] using both qualitative examination and calibration curves reported in Figure 9a, we observed that the citrate-coated BaGdF_5_: 10% Eu^3+^ nanoparticles outperformed the contrast capability of the commercial iodine-based Optiray-350 agent. The obtained contrast superiority was due to the fact that Ba and Gd more readily absorb high-energy X-ray photons having a larger X-ray attenuation coefficient compared with I (at 60 kV, Ba = 8.51, Gd = 11.75 and I = 7.58 cm^2^ g^−1^) [54].

Biodistribution of the citrate-coated BaGdF_5_: 10% Eu^3+^ nanoparticles were assessed through in vivo CT imaging mice after an intravenous injection of 200 µL nanoparticle aqueous solution (total dosage of Gd, ca. 2.9 mg as estimated considering the TGA curve of Cit^3−^-coated NPs, see Figure S3). Figure 9c demonstrates a series of CT images acquired before the injection and at different time intervals after the injection (some contrast regions in the gastrointestinal area were observed due to the residual amount of animal feed).

From the visual examination, one observes that just after the nanoparticles’ administration (5 min), significant contrast enhancement of the spleen was detected, and the border of the liver became better detectable compared with the pre-injection visualization. Comparison of the CT scans taken after 30 min and 2 h demonstrates continuous contrast increase in the liver and the spleen (more substantially). In addition, nanoparticle biodistribution was quantitatively estimated by analyzing the contrast for ROI, which corresponds to different mouse organs (liver, spleen, kidney and heart were considered). This analysis confirmed the previously observed trends (see Figure 9b). In more detail, just after contrast administration, a notable HU increase was observed for all the organs, except for kidneys where the contrast level remained almost unchanged after different time intervals probed. Interestingly, for the heart, some short-term threshold of contrast enhancement was detected (5 min CT), which was further notably reduced. As for the liver, a more than double HU enhancement was observed, and the nanoparticle concentration in the liver peaked at 1–2 h after the injection, with subsequent smooth decline. Finally, the greatest accumulation effect was observed for the spleen. Compared with the liver, a more pronounced contrast increase was detected with 30 min delay after the injection and remained almost unchanged for the next few hours (1 h and 2 h scan). However, a further notable HU increase was obtained after 24 h, thus demonstrating quite pronounced nanoparticle accumulation in the spleen.

The obtained results are somewhat different from the ones recently reported by Wang et al. [52] where CT-based biodistribution of RGD-loaded PEGylated nanoparticles was reported for tumor-bearing mice and dominating nanoparticle accumulation was shown in the liver. The observed difference might have been due to different reasons. First, in their work, a well-defined contrast increase was observed for tumor tissues as well. Second, despite the pretty similar size of nanoparticle cores (ca. 9–10 nm in [52] vs. 14–15 nm in our work), the different coating and presence of an RGD agent (that likely resulted in the different values of ζ-potentials) could significantly affect nanoparticle biodistribution. The metabolic behavior of nanoparticles in vivo depends not only on their own characteristics, such as size, morphology, charge, surface functional groups, aspect ratio and so on, but also on the tissue microenvironment [52].

Overall, the observed NP accumulation in the liver and the spleen is typical since both organs are characterized by a well-developed vascular system, while the prolonged effect of NP accumulation in the spleen will be addressed in our further work via in vivo toxicity experiments.

### 2.3. SiO_2_ Surface Modification

Since the luminescent profiles of the BaGdF_5_: 10% Eu^3+^ sample were the most intensive (according to Figure 5a), the Eu^3+^-doped nanophosphors were coated with SiO_2_ for further impregnation of photosensitizer molecules in the silica porous structure. The coated nanoparticles were investigated by X-ray diffraction, X-ray fluorescence, transmission electron microscopy, automated surface area and the porosimetry (ASAP) method.

#### 2.3.1. Structure and Morphology

**Figure 10:**
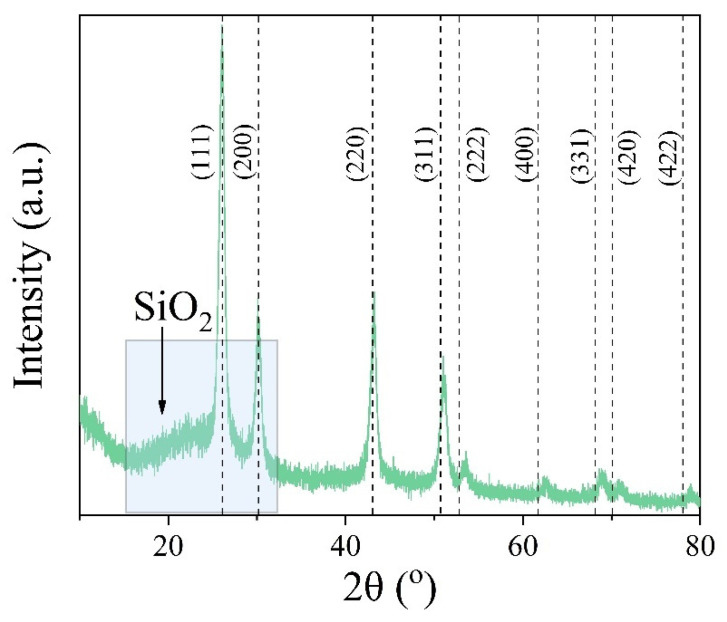
X-ray diffraction pattern of the BaGdF_5_: 10% Eu^3+^@SiO_2_ sample.

Qualitative analysis of the X-ray powder diffraction profile confirms that after SiO_2_ coating, the core of a nanocomposite preserves a single-phase BaGdF_5_ structure. Moreover, an additional broad peak centered around 2θ = 25° was observed (Figure 10). This peak could be assigned to the SiO_2_ amorphous microspheres [55].

**Figure 11:**
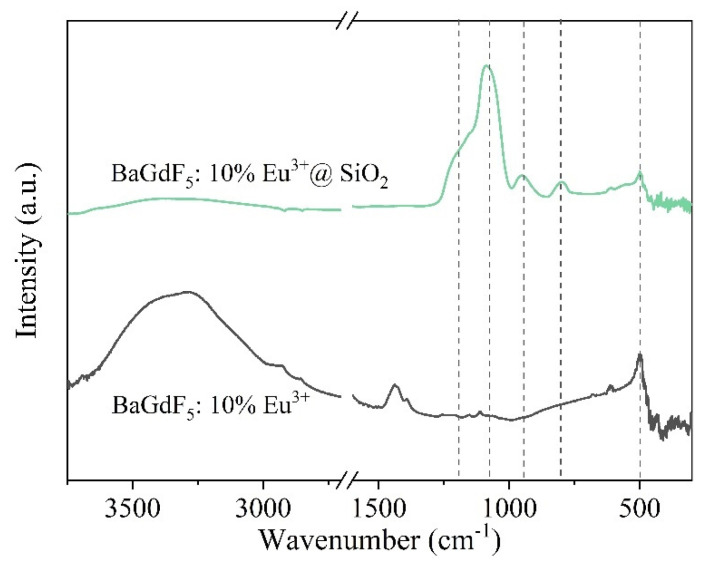
FTIR spectra of BaGdF_5_: 10% Eu^3+^@SiO_2_ and BaGdF_5_: 10% Eu^3+^.

The Fourier-transform infrared spectra were measured for the SiO_2_-coated samples to detect amorphous silica. According to the FTIR spectra in Figure 11, the bands at 801 cm^−1^ and 498 cm^−1^ relate to the Si–O bond bending and bond rocking vibrations in the three-dimensional SiO_2_ network [56]. The shoulder at 944 cm^−1^ is caused by the Si–O and Si–OH bonds on the silica shell surface [57]. The most intense band at 1077 cm^−1^ with a shoulder at 1187 cm^−1^ is attributed to the transverse optical and longitudinal optical modes of Si–O–Si asymmetric stretching vibrations [58]. Thus, FTIR confirms formation of the amorphous SiO_2_ structure in the BaGdF_5_: 10% Eu^3+^@SiO_2_ sample.

**Figure 12:**
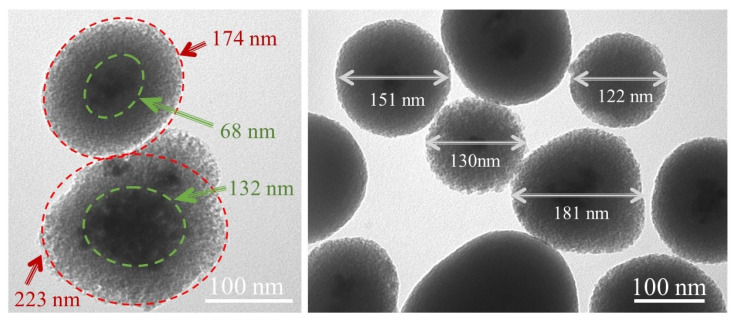
TEM imaging measurements of BaGdF_5_: 10% Eu^3+^@SiO_2_.

Finally, according to the TEM images of the SiO_2_-coated samples (Figure 12), the average size of the BaGdF_5_: 10% Eu^3+^@SiO_2_ core–shell nanoparticles was estimated as 164 nm, the width of the coating ~119 nm, the width of the core ~45 nm. The core size increase was due to the agglomeration of nanoparticles under synthesis conditions. Thus, even after the SiO_2_ coating, the obtained composites overcome biological barriers [26], the nanospheres can be truly transported through the capillary system.

#### 2.3.2. Tunable SiO_2_ Coating

Next, the amount of SiO_2_ absorbed on the surface of nanoparticles was estimated from XRF analysis. The obtained results demonstrate that the content of Si was about 60 at. %. Obviously, too thick an amorphous SiO_2_ shell might be a serious problem for re-emitted light propagation and may substantially inhibit the Förster resonance energy transfer (FRET) mechanism which is known to be limited by 5–10 nm [59,60,61]. On the one hand, according to the Brunauer–Emmett–Teller (BET) surface analysis reported below, amorphous SiO_2_ can be considered as a mesoporous material and PS molecules can be located inside the pores in the proximity of scintillating nanoparticles. On the other hand, for ROS generation, PS molecules must be easily achievable for molecular oxygen, while this process can be partially inhibited by the thick amorphous silica shell.

Taking into account the abovementioned considerations, we demonstrated the possibility of tunable shell thickness using two different routes: (i) varying the treatment time (10, 20, 30 min) and keeping fixed the amount of tetraethyl orthosilicate (TEOS) (325 µL) and (ii) with a fixed treatment time (40 min) and varied TEOS amount (TEOS was reduced by two, four, six and eight times). A similar approach was recently reported for LaF_3_:Tb nanoparticle coating by Elmenoufy et al. [62].

**Figure 13:**
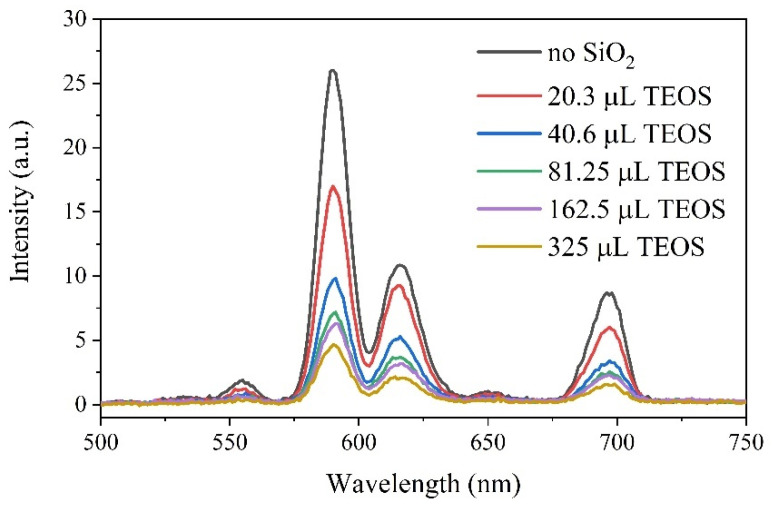
XEOL signal collected for the SiO_2_-coated BaGdF_5_: 10% Eu^3+^ nanoparticles obtained by varying the amount of TEOS.

The amount of SiO_2_ was subsequently measured with XRF. The first method resulted in ca. 55–56 at. % of the Si content independently of the TEOS–nanoparticles interaction time (see Figure S4a). At the same time, for the method based on different TEOS loading, very good correspondence between the introduced TEOS amount and the measured Si content was obtained (see Figure S4b). Moreover, XEOL spectra acquired for the set of SiO_2_-coated samples obtained using different amounts of TEOS demonstrated the gradual decrease of the signal intensity with the increase in TEOS loading upon surface coverage (Figure 13).

The observed trend in the registered XEOL intensity demonstrates an explicit exponential character (as shown in Figure S5 in the Supplementary Materials). In addition, the fact that after the interaction with TEOS the system was centrifuged at high rpm and thoroughly washed, the Si content data obtained from XRF and XEOL signal attenuation allowed us to declare the formation of SiO_2_-coated nanoparticles with varied thickness of the SiO_2_ shell.

For the latter N_2_ adsorption–desorption isotherms characterization and methylene blue conjugation, the sample with larger SiO_2_ layer thickness was selected in order to get a more representative effect of SiO_2_ coating.

#### 2.3.3. Measurements of the Specific Surface Area and Pore Size Distribution of the Silica Shell Structure

After coating the samples with SiO_2_, the surface area and pore size of the nanostructures were verified. The isotherms of the samples can be attributed to type IV (IUPAC classification), which is typical of mesoporous materials. Indeed, the most probable size of the BaGdF_5_: 10% Eu^3+^@SiO_2_ nanoparticles was 164 nm according to the TEM analysis (Figure 12). The size of pores corresponded to mesoporous nanostructures (according to the IUPAC classification).

**Figure 14:**
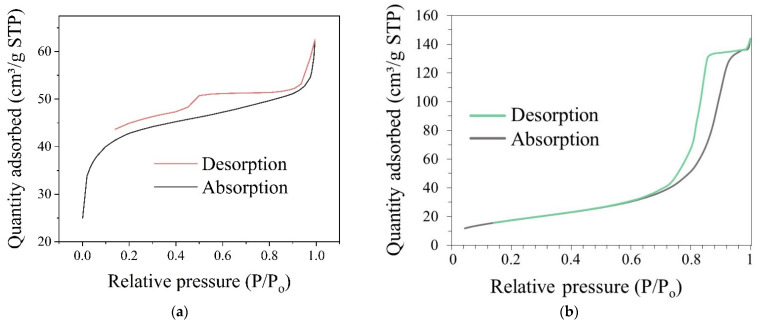
Adsorption, desorption isotherms of the nitrogen adsorption test of (**a**) BaGdF_5_: 10% Eu^3+^@SiO_2_, (**b**) BaGdF_5_: 10% Eu^3+^.

The phenomenon of hysteresis in the isotherms of low-temperature nitrogen adsorption is related to capillary condensation in mesoporous structures. Different types of adsorption and sorption environments (temperature and pressure) affect the hysteresis loop shapes of the investigated samples. In the hysteresis loop of the BaGdF_5_: 10% Eu^3+^@SiO_2_ sample (Figure 14a), the shape of the pore corresponds to the H3 type of hysteresis. H3 is associated with the slit-shaped type of pores. Thus, the BaGdF_5_: 10% Eu^3+^@SiO_2_ nanoparticles had slit-shaped pores. This kind of pores assumes that it should be possible to impregnate several photosensitizer molecules in the pores. The desorption curve of H3 hysteresis contains a slope related to the force on the hysteresis loop due to the so-called tensile strength effect (this phenomenon probably occurs for nitrogen at 77 K in the relative pressure range from 0.4 to 0.45).

In comparisons with the noncoated sample (Figure 14b), the type of adsorption isotherms changed. The isotherms of the noncoated sample can be attributed to type III (IUPAC classification), which is typical of nonporous or macroporous materials. Indeed, according to the Barrett–Joyner–Halenda (BJH) calculations, the pore size was 11.9 nm and the surface area was 64 m²/g. The obtained value was in good correspondence with an averaged interparticle distance estimated as 15 nm from TEM images (see Figure 4d). Moreover, the noncoated nanoparticles could be attributed to type H1 associated with capillary condensation of nitrogen in spaces between uniform nanoparticles in agglomerates.

**Figure 15:**
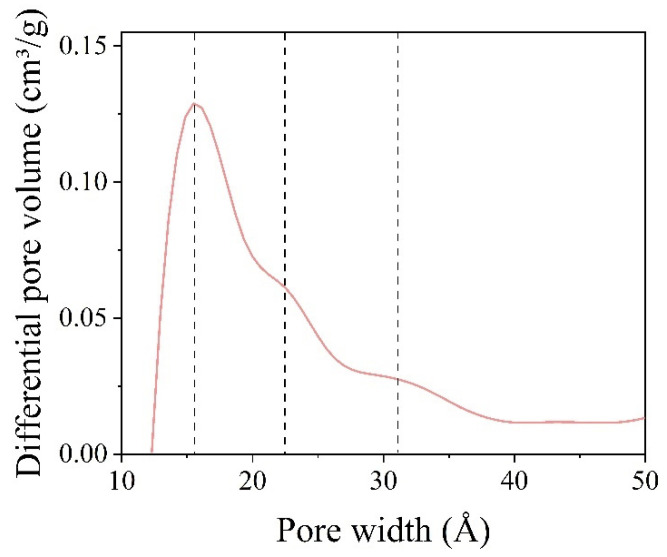
Pore diameter logarithm differential distribution graph for BaGdF_5_: 10% Eu^3+^@SiO_2_ calculated from the nitrogen absorption data.

Turning back to the coated nanoparticles, the sizes of pores were obtained from the pore diameter logarithm differential distribution graph of BaGdF_5_: 10% Eu^3+^@SiO_2_ by means of nitrogen adsorption (see Figure 15). The nonlocal density functional theory (NLDFT) model was used to determine the porosity of a sample—pore width. According to NLDFT, the pore sizes were 1.5 nm, 2.3 nm and 3.1 nm. Furthermore, according to the BJH calculations, the pore size was estimated as 4.26 nm, the pore volume was 0.03 cm³/g and according to the Brunauer–Emmett–Teller theory, the surface area was 160.11 m²/g.

#### 2.3.4. Cytotoxicity Test

**Figure 16:**
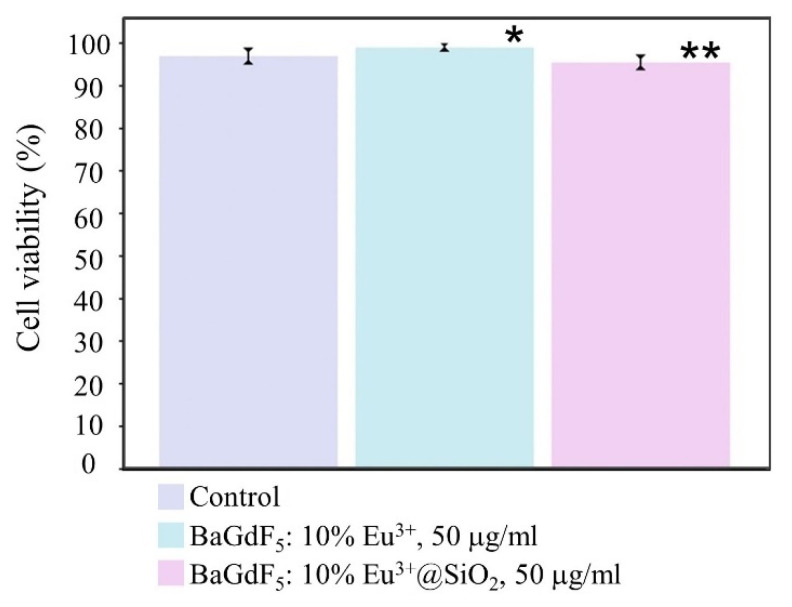
HeLa cells’ viability following exposure to saline (control group), 50 μg/mL BaGdF_5_: 10% Eu^3+^ and 50 μg/mL BaGdF_5_: 10% Eu^3+^@SiO_2_ for 24 h with a subsequent trypan blue exclusion assay. Note: * *p* < 0.02 when compared to the control; ** *p* < 0.001—differences are significant between the nanocomposite groups. The Kruskal–Wallis criterion testing returned *p* = 0.001.

In addition to physicochemical characterization of the nanocomposites, we assessed their comparative effect on the HeLa cells’ viability (Figure 16).

As seen from the collected data, the nanocomposites notably differed in their effects. The noncoated variant, BaGdF_5_: 10% Eu^3+^, caused an approximately 3% increase in cell viability. We would speculate that these effects might be due to the redox properties of the nanocomposite increasing cell adaptation capabilities because of the respective signaling changes and thanks to the elemental composition of the nanoagent [63]. This, in turn, perfectly explains why the coated counterpart did not have this effect: silica coating is known to hamper nanocomposite degradation, thus probably preventing the emanating ions from causing any significant shifts in the oxidative status of the cells. This hypothesis will be tested in our future research.

As for the minor (~1%) yet significant decrease in viability in the silica-coated nanoparticle group, when compared to the control group, although these changes were detected statistically, they were nonrelevant biologically. The decrease might have been due to the silica coating itself since it is not entirely inert [63], but this minimal magnitude of the reaction would render the respective investigation of the cause cost-ineffective.

To conclude on the biological part of characterization, both formulations demonstrated a perfect safety profile, with silica coating introducing an emergent biological influence on cell viability. This influence was most likely due to physicochemical properties and surface features of the amorphous silica layer itself and the decreased rare element-containing core degradation rate.

#### 2.3.5. Nanocomposite Impregnation by the Methylene Blue Photosensitizer

The construction of a nanocomposite for efficient XPDT remains an ongoing challenge since several important aspects must be satisfied such as good biocompatibility and toxicity, efficient energy transfer from scintillating NPs and PS molecules, close proximity of molecular oxygen, etc. For an efficient energy transfer, two aspects are of crucial importance: (i) the FRET mechanism strongly depends on the spacing between the emitting sites and the PS molecules [59,60,61] and (ii) the emission spectrum of nanophosphors must at least partially overlap the absorption spectrum of the photosensitizer. The last criterion makes composite construction based on Eu^3+^-doped NPs particularly challenging due to the lack of suitable PS dyes.

Thereby, Chouikrat et al. [64] reported the design of europium-doped composite GdO_2_S:Eu^3+^ conjugated with zinc chlorin (ZnTPC) and zinc phthalocyanine (ZnPc). It has been shown that under direct excitation, PSs were capable of producing ^1^O_2_, while when NPs were excited by UV light or X-rays, no efficient energy transfer was observed. An efficient energy transfer and subsequent ROS generation were reported by Chen et al. [65] for a composite based on SiO_2_-coated SrAl_2_O_4_:Eu^2+^ NPs. Bright green light emission (with a maximum at ca. 520 nm) allowed using the MC540 dye for this composite. Generally, the hypericin dye provides an almost perfect overlap with the typical emission spectrum of Eu^3+^ but it is unstable at room temperature without additional treatment [66,67]. Nevertheless, Kascakova et al. [67] declared ^1^O_2_ ROS generation upon X-ray irradiation for hypericin-conjugated GdEuC_12_ micelles. Finally, Yefimova et al. [38] investigated energy transfer for GdVO:Eu^3+^ NPs conjugated with different cationic dyes DiOC2, DiDC1 and methylene blue. The FRET efficiency for MB conjugation has been estimated at 90% and in the latter work by the same group [67], efficient ROS generation for GdVO:Eu^3+^@ MB was demonstrated under X-ray irradiation.

**Figure 17:**
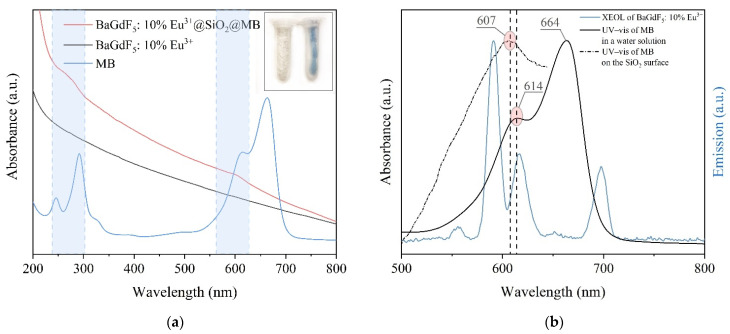
(**a**) UV–vis spectra of BaGdF_5_: 10% Eu^3+^@SiO_2_@MB, BaGdF_5_: 10% Eu^3+^, MB; (**b**) UV–vis absorption spectra of MB in a water solution, MB on the nanoparticles’ surface and the XEOL emission spectrum of BaGdF_5_: 10% Eu^3+^.

Thus, at the next stage, we prepared a composite based on SiO_2_-coated and uncoated BaGdF_5_: 10% Eu^3+^ conjugated with the methylene blue dye, known as an efficient photosensitizer for different applications [68,69]. The emission spectrum of BaGdF_5_:Eu^3+^ at least partially overlaps the optical absorption of MB (see Figure 17a), thus making the latter suitable for nanocomposite construction.

For quantification, we first performed calibration using a stock MB solution with different concentrations (2, 4 and 6 µg/mL). The corresponding UV–vis spectra obtained for stock solutions and mother liquors are reported in Figure S6.

We observed that the mother liquor (ML) obtained after SiO_2_-coated nanoparticle impregnation had a notably smaller amount of residual MB molecules compared with those obtained after noncoated nanoparticles. For further quantification, we used the obtained calibration curve which maintained the correspondence between the MB concentration and the intensity of the signal at 664 nm (see the inset in Figure S6).

Thus, the sorption capacity was approx. 0.54 and 1.1 μg MB per 1 mg of nanoparticles and SiO_2_-coated nanoparticles, respectively. Considering that the averaged size of SiO_2_-coated nanoparticles used for the impregnation experiment (ca. 164 nm) was notably larger compared with those without SiO_2_ coating (ca. 14 nm) as well as the mutual repulsion of positively charged colloidal BaGdF_5_: 10% Eu^3+^ and positively charged MB molecules, we can declare that SiO_2_ coating, due to the surface area increase and negative ζ-potential, can significantly enhance the sorption capacity of the MB photosensitizer.

From the UV–vis spectra of the MB-impregnated and nonimpregnated samples (see Figure 17a), we can see that the sample impregnated with MB had broad low-intensity peaks characteristic of the photosensitizer. For greater clarity, we subtracted the BaGdF_5_: 10% Eu^3+^ spectrum from the BaGdF_5_: 10% Eu^3+^@SiO_2_@MB spectrum and obtained a more accurate resulting adsorption peak position (dash–dot plot in Figure 17b). It is well-known [70] that aqueous solutions of methylene blue have two main absorption peaks at 664 and 614 nm, corresponding to the monomeric and dimeric dye forms, respectively. However, we can also observe a hypsochromic shift of the main absorption peaks to 607 nm. This shift may indicate the binding of methylene blue molecules to the surface of nanoparticles, as well as the formation of so-called H-aggregates [71]. H-aggregates of methylene blue are dimers in which phenothiazine rings are stacked plane-to-plane, resulting in a π–π (stacking) interaction of two molecules. For methylene blue, trimers and tetramers are also known. The presence of the dimeric form is confirmed by the resulting absorption peak wavelength (approx. 607 nm) [72]. The formation of H-dimers on the silica surface has already been discussed earlier [73]. The predominance of the dimeric form as well as its absorption peak’s hypsochromic shift leads to a more efficient UV–vis spectrum overlaps with the XEOL spectrum (Figure 17b). This contributes to a more efficient transfer of radiation from the nanophosphor to the photosensitizer.

## 3. Discussion

Thus, the BaGdF_5_: x% Eu^3+^ (x = 1, 2.5, 5, 10, 25, 50) nanophosphors, BaGdF_5_: 10% Eu^3+^@SiO_2_, BaGdF_5_: 10% Eu^3+^@SiO_2_@MB and BaGdF_5_: 10% Eu^3+^@Cit^3−^ were synthesized. TEM imaging demonstrated the formation of monodispersed NPs with the average size ~12 nm and narrow size distribution for all Eu:Gd ratios. According to the XEOL spectra, the BaGdF_5_: 10% Eu^3+^ nanophosphors demonstrated the most intensive luminescence. For this reason, the 10% doped NPs were selected for subsequent surface modification with a citrate and silica_._ Citrate coating results in improved colloidal solution stability while SiO_2_ substantially facilitates methylene blue conjugation upon BaGdF_5_: 10% Eu^3+^@SiO_2_@MB nanocomposite construction. Moreover, we showed a possibility of coating with tunable SiO_2_ layer thickness which is important for optimization of the energy transfer between scintillating NPs and PS molecules. The synthesized nanostructures can be used as X-ray-activated agents in an XPDT system. In addition, BaGdF_5_: 10% Eu^3+^@Cit^3−^ nanophosphors have been also shown as an efficient contrast agent for CT visualization.

## 4. Materials and Methods

### 4.1. Characterization Techniques

XRD of the synthesized nanoparticles was measured by means of D2 PHASER using Cu K_α_ radiation (λ = 1.5406 Å) at 30 kV and 10 mA. For the measurements, we used a low-background cuvette and the following parameters: 2θ range from 5° to 90°, step size—0.01°.

An FEI Tecnai G2 Spirit BioTWIN was used to perform TEM for imaging of the obtained samples. An accelerating voltage of 80 kV was used.

Qualitative and quantitative element analysis of the synthesized nanoparticles was carried out using XRF spectrometer M4 Tornado. The data were collected with 20–25 points of a sample surface, 10 s acquisition for each. Thermogravimetric analysis (TGA) was performed using thermal gravimetric analyzer STA 449 F5 Jupiter (Netzsch). Corundum crucibles for measurement in the flux of air with a heating rate of 10 °C/min were employed.

An XEOL signal was detected by using an in-house setup based on an Agilent Cary Eclipse fluorescence spectrophotometer with the emission slit set to 10 nm and an X-ray tube. The following parameters of the X-ray tube were applied for XEOL registration: voltage of 35 kV, current of 1.6 mA. Powder samples were thoroughly grinded and deposited on a thin film transparent both for optics and X-rays. Afterwards, the sample was placed under X-rays maintaining the angle of 45° between the sample surface and both incident X-ray beams and the fluorescence detector.

Both in vitro and in vivo imaging was performed on a Quantum GX-2 micro-CT device (Perkin Elmer, Boston, MA, USA). For in vitro CT imaging, an aqueous solution of BaGdF_5_: 10% Eu^3+^ and commercially available nonionic contrast agent Optiray^®^ (350 mg (I)/mL) in different concentrations were prepared and placed in Eppendorf tubes in a 3D-printed plastic sample holder for positioning inside a micro-CT chamber and the following CT acquisition parameters were employed: tube voltage = 80 kV, tube current = 90 µA, scan time = 2 min. Contrast capabilities were determined in Hounsfield units.

For the in vivo CT measurement, intact BALB/c male mice (ca. 3 months old, 34–35 g) were anesthetized with 2% isoflurane (Laboratories Kari-zoo, S.A., Barcelona, Spain) using a dedicated RAS-4 anesthesia device (Perkin Elmer, USA). An 89 mM aqueous solution of citrate-coated BaGdF_5_: 10% Eu^3+^ (200 µL, ca. 7 mg of nanoparticles) was administrated intravenously. After the different time intervals, micro-CT images were taken with the following parameters: tube voltage = 80 kV, tube current = 90 µA, FOV was restricted to an 86 × 72 mm rectangle, voxel size = 140 µm. For each CT scan, acquisition time was equal to 4 min, corresponding to a radiation dose of 136 mGy. All the animal experiments were carried out in accordance with the Guide for the Care and Use of Laboratory Animals [74].

Fourier-transform infrared spectra were measured on a Bruker Vertex 70 spectrometer in ATR (attenuated total reflectance) geometry using an MCT detector and a Bruker Platinum ATR attachment. The spectra were measured in the range from 5000 to 300 cm^−1^ with a resolution of 1 cm^−1^. Sixty-four scans were collected and averaged for each measurement. Air at RT was utilized as the reference.

Hydrodynamic particle size distribution data were measured using a NANO-flex particle size analyzer. The data were accumulated over five consecutive measurements of 2 min each and then summarized considering temperature, dynamic viscosity, particle concentration and many other parameters that affect the result. The signal collected for a pure solvent (water) was subtracted as the background. The measurements were performed for 10 mL of the sample solution with the concentration of nanoparticles of 1 mg/mL. Before the measurement, the solution was stirred for 40 min.

Rapid particle charge titrations and size distribution analysis of the samples were carried out using Stabino. The data were collected using the mode of statistics accumulation for 24 points with an interval of 5 s. The solution was preliminarily stirred for two minutes with the concentration of the substance in water of 2 mg/mL.

Nitrogen adsorption–desorption isotherms were measured at −196 °C obtained on accelerated surface area and porosimetry analyzer ASAP 2020 (Micromeritics Instruments Corp., Norcross, GA, USA). The samples were activated at 250 °C for 10 h under dynamic vacuum before the measurement.

The UV–vis spectra of photosensitizers were obtained with Shimadzu UV-2600. The spectra were measured in the range from 190 to 900 cm^−1^ with a resolution of 1 cm^−1^. 

### 4.2. Synthesis Technique of the BaGdF_5_: x% Eu^3+^ Nanophosphors

The microwave method developed by our scientific group and previously described in [75] was chosen as the synthesis technique. A series of ultra-small BaGdF_5_: x% Eu^3+^ nanoparticles were obtained with different concentrations of Eu (i.e., x = 1, 2.5, 5, 10, 25, 50—the EuCl_3_ percentage relative to the GdCl_3_ molar amount). The amounts of precursors used in the synthesis and sample marking are presented in Table 3.

First, a mixture of GdCl_3_ and EuCl_3_·6H_2_O was dissolved in 20 mL of ethylene glycol by ultrasonic treatment. Then, BaCl_2_·2H_2_O was added to the resulting solution and sonicated until complete dissolution, after which the solution was stirred with a magnetic stirrer for 30 min. After that, 1.5 g of PEG-1500 was dissolved in the solution under ultrasonic treatment for 15 min. Separately, a solution of NH_4_F in 10 mL of ethylene glycol was prepared. Then, the two solutions were combined, sonicated for 5 min, transferred to a Teflon ampoule and placed in MW oven CEM Mars6 (microwave-accelerated reaction system). The microwave synthesis conditions were as follows: rise time = 20 min, temperature = 200 °C, temperature holding time = 2 h, with medium mixing of the solution and power of 600 W. After that, the mixture was centrifuged (13,000 rpm for 10 min) twice with water. The resulting solution was dried at 60 °C.

### 4.3. Citrate Coating

Sodium citrate (0.7 g) was dissolved in 50 mL deionized water; 0.06 g of BaGdF_5_: 10% Eu^3+^ was added to this solution and the mixture was ultrasonicated at 50 °C for 30 min instead of 1 h as described in [76]. White-colored nanoparticles were washed several times with deionized water and then with acetone to remove excess citrate groups. The obtained nanoparticles were dried for 24 h at 60 °C. The synthesized sample was marked as BaGdF_5_: 10% Eu^3+^@Cit^3−^.

### 4.4. SiO_2_ Coating

All the synthesized BaGdF_5_: x% Eu^3+^ (x = 1, 2.5, 5, 10, 25, 50) samples were coated with SiO_2_. The preparation of the composites was carried out as follows: 100 mg of BaGdF_5_: x% Eu^3+^ (x = 1, 2.5, 5, 10, 25, 50) were added to a 25 mL aqueous solution of polyvinylpyrrolidone (concentration, 1 mg/mL). The obtained mixture was kept under ultrasonication to obtain complete dissolution. After that, the solution was centrifuged (13,000 rpm for 10 min) twice in methanol. Then, the centrifuging precipitate was transferred to 20 mL of methanol, 10 mL of a concentrated ammonia solution, and 325 µL of TEOS were added to the solution. The solution was left stirring with a magnetic stirrer for 24 h. After that, the mixture was centrifuged (13,000 rpm for 10 min) twice with water. The resulting solution was dried at 60 °C for 24 h.

To vary the thickness of the SiO_2_ shell, two approaches were used: varying the synthesis time and varying the amount of TEOS. In the first approach, particles during the synthesis were sampled every 10 min for 1 h. In the second approach, a series of four syntheses was carried out, but the amount of TEOS was reduced by two, four, six and eight times, respectively.

### 4.5. Cytotoxicity Test

In this study, HeLa cells were used as the in vitro experimental model for the cytotoxicity test. The cells were seeded and cultured in 24-well plates (SPL LifeSciences, Pocheon, Korea) in the GlutaMax DMEM medium (Thermo Fisher Scientific, Waltham, MA, USA) supplemented with 10% fetal bovine serum (GE Healthcare, Chalfont St Giles, UK), 50 IU/mL penicillin and 50 µg/mL streptomycin (Thermo Fisher Scientific, Waltham, MA, USA). The cells were incubated at 37 °C and 5% CO_2_ in a Binder CB-150 incubator (Binder, Tuttlingen, Germany). Monolayer formation was observed, and common assessment of the cell culture was performed using a Premiere MIS-9000 inverted microscope (C&A, Shanghai, China).

To assess cytotoxicity of the nanoagents, we performed a trypan blue exclusion assay using automated cell viability analyzer Countess II FL and the protocol of the manufacturer (Thermo Fisher Scientific, Waltham, MA, USA). During the experiment, stock solutions of nanomaterials in saline were introduced into the culture medium at the concentration of 50 μg/mL. In the control group, saline was added to the medium. Following addition of the test samples, the cells were incubated for 24 h.

### 4.6. Nanoparticle Impregnation with the Methylene Blue Photosensitizer

We prepared 15 mL of a methylene blue solution (concentration, 8 μg/mL (2.5 × 10^−5^ M)). The UV–vis spectra of this solution were measured. The next two vessels were filled with 15 mL of the MB stock solution and 50 mg of BaGdF_5_: 10% Eu^3+^ and BaGdF_5_: 10% Eu^3+^@SiO_2_ were added, and the obtained mixture was stirred for 1 h at room temperature. Then, the precipitate was separated from the solution by centrifugation, and the absorption spectrum of the supernatant was measured. The precipitate was washed three times with distilled water and dried at 60 °C, and the obtained samples were marked as BaGdF_5_: 10% Eu^3+^@MB and BaGdF_5_: 10% Eu^3+^@SiO_2_@MB.

## Figures and Tables

**Table 1:** Cell parameters of the BaGdF_5_ and BaGdF_5_: x% Eu^3+^ samples calculated from full profile analysis in the Jana2006 software and crystal size calculated using the Scherrer equation and the Williamson–Hall analysis.

Sample Names	Initial Elemental Composition at., %	Actual Elemental Composition at., %	Cell Parameters, Å	Cell Volume, Å^3^	Crystal Size According to the Scherrer Equation	Crystal Size According to the W–H Analysis
BaGdF_5_	0	0	5.9099 (5)	206.41 (3)	10.71	14.20
BaGdF_5_: 1% Eu^3+^	0.14	0.02	5.9198 (8)	207.45 (3)	10.45	13.59
BaGdF_5_: 2.5% Eu^3+^	0.36	0.20	5.9168 (8)	207.13 (8)	10.49	14.20
BaGdF_5_: 5% Eu^3+^	0.71	0.50	5.9208 (14)	207.55 (8)	9.64	11.47
BaGdF_5_: 10% Eu^3+^	1.43	1.37	5.9212 (7)	207.60 (1)	10.98	15.36
BaGdF_5_: 25% Eu^3+^	3.57	3.51	5.9276 (9)	208.27 (4)	9.61	12.64
BaGdF_5_: 50% Eu^3+^	7.14	8.33	5.9388 (8)	209.45 (5)	9.99	12.21

**Table 2:** Ζ-potential measurements for the BaGdF_5_ and the BaGdF_5_: x% Eu^3+^ samples.

Sample	ζ-Potential, mV
BaGdF_5_	26.12
BaGdF_5_: 1% Eu^3+^	29.89
BaGdF_5_: 2.5% Eu^3+^	32.04
BaGdF_5_: 5% Eu^3+^	25.16
BaGdF_5_: 10% Eu^3+^	32.37
BaGdF_5_: 25% Eu^3+^	25.49
BaGdF_5_: 50% Eu^3+^	28.57

**Table 3:** Amounts of precursors used in the synthesis process.

Sample	BaCl_2_·2H_2_O		GdCl_3_		EuCl_3_·6H_2_O		NH_4_F	
	mg	mmol	mg	mmol	Mg	mmol	mg	mmol
BaGdF_5_: 1% Eu	244.3	1S	261	0.99	3.7	0.01	203.7	5.5
BaGdF_5_: 2.5% Eu			257	0.975	9.2	0.025		
BaGdF_5_: 5% Eu			250.4	0.95	18.3	0.05		
BaGdF_5_: 10% Eu			210.9	0.8	36.6	0.1		
BaGdF_5_: 25% Eu			197.7	0.75	91.6	0.25		
BaGdF_5_: 50% Eu			131.8	0.5	183.2	0.5		

## Data Availability

All the data are contained in the manuscript and its Supplementary Materials.

## Supplementary Materials

The following are available online at https://www.mdpi.com/article/10.3390/ijms222313040/s1.
