# Supplementary material for: BaGdF5 Nanophosphors Doped with Different Concentrations of Eu3+ for Application in X-ray Photodynamic Therapy
Source: Int J Mol Sci. 2021 Dec 2;22(23):13040. doi: 10.3390/ijms222313040 (PMC8657490; doi:10.3390/ijms222313040)
Supplement: Supplementary file 1 [file ijms-22-13040-s001.zip › ijms-1479080-supplementary.pdf]

International Journal of
Molecular Sciences

MDPI

Supplementary Materials:

- Table S1: Calculated initial elemental composition of the $BaGdF_5$:x%Eu^{3+} samples and elemental composition from X-ray fluorescence measurements,
- Figure S1: FTIR spectra of $BaGdF_5$:x%Eu^{3+} (x = 1, 2.5, 5, 10, 25, 50),
- Figure S2: TGA (a) and DSC (b) curves of PEG-coated and uncoated particles,
- Figure S3: TGA curve obtained for $BaGdF_5$:10%Eu^{3+}@Cit^{3-} nanoparticles taken upon nitrogen atmosphere,
- Figure S4: Si at. % determined from XRF for $BaGdF_5$:10%Eu^{3+} sample as obtained after two different SiO_2 method: (a) with fixed amount of loaded TEOS and varied interaction time and (b) with fixed interaction time and varied amount of TEOS,
- Figure S5: The intensity of XEOL peaks intensity at 590 (a) and 697 nm (b) obtained for the series of $BaGdF_5$:10%Eu^{3+} nanophosphors coated with different amount of SiO_2. The red curves correspond to the trend lines obtained via standard exponential decay fit,
- Figure S6: UV-vis absorbance spectra obtained for a series of stock MB solutions with different concentration and for mother liquors collected after nanoparticles impregnation with MB solution (8 μg/ml). Dashed orange and green curves correspond to mother liquors obtained for nanoparticles and SiO_2-coated nanoparticles impregnation, respectively. The inset demonstrates calibration curve for quantification of absorbed MB.

Table S1. Calculated initial elemental composition of the $BaGdF_5$:x%Eu^{3+} samples and elemental composition from X-ray fluorescence measurements.

Sample names	Initial elemental composition at. %				Actual elemental composition at. %			
	Ba	Gd	Eu	F	Ba	Gd	Eu	F
$BaGdF_5$	14.29	14.29	0	71.43	11.89	16.08	0	72.03
$BaGdF_5$:Eu^{3+} 1%	14.29	14.14	0.14	71.43	11.48	16.37	0.02	72.13
$BaGdF_5$:Eu^{3+} 2.5%	14.29	13.93	0.36	71.43	11.53	16.15	0.20	72.12
$BaGdF_5$:Eu^{3+} 5%	14.29	13.57	0.71	71.43	11.51	15.87	0.50	72.12
$BaGdF_5$:Eu^{3+} 10%	14.29	12.86	1.43	71.43	11.64	14.90	1.37	72.09
$BaGdF_5$:Eu^{3+} 25%	14.29	10.71	3.57	71.43	12.09	12.42	3.51	71.98
$BaGdF_5$:Eu^{3+} 50%	14.29	7.14	7.14	71.43	12.46	7.33	8.33	71.88

The purity of the final products was monitored by Fourier-transform infrared spectroscopy (FTIR) spectroscopy (Figure S1). All samples demonstrate similar IR profile according to the [1,2]. The broad peak at 3500-3000 cm^{-1}, as well as at 1643 cm^{-1}, corresponds to the stretching and bending vibrations of water molecules adsorbed on the surface of the nanoparticles. The peak at 1442 cm^{-1} corresponds to the stretching vibrations of the Gd-F and Ba-F bonds [1]. The peak at 1053 cm^{-1} corresponds to O-H bonds of primary alcohol groups [2]. We can also observe a low-intensity peak at 2964 cm^{-1}, associated with vibrations of the isopropanol methyl groups, which was used to wash the device before measurements.

It should be noted that several peaks on the FTIR spectra reveal PEG molecules present on the nanoparticles surface. The peak at 1092 cm^{-1} can be assigned with bonds of PEG chains and two low-intensity peaks at 2949 cm^{-1} and 2878 cm^{-1} corresponds to asymmetric and symmetric CH_2 stretching modes. The obtained results are consistent with those reported in the literature [3-5].

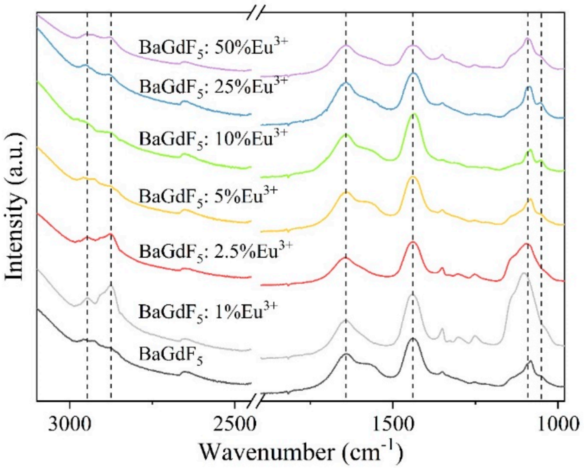

Figure S1. FTIR spectra of $BaGdF_5$: x% Eu^{3+} (x = 1, 2.5, 5, 10, 25, 50).

When comparing the Thermogravimetric analysis (TGA) curves of PEG-coated and uncoated particles, a slight difference in weight loss can be seen (Figure S2a). This difference, apparently, is the fraction of PEG molecules on the NPs surface. Differential Scanning Calorimetry (DSC) shows two peaks of heat release at 320 and 430 ˚C of the same shape for both samples (Figure S2b). This fact indicates the relatedness of the thermal decomposition processes. Probably, the heat release peaks correspond to two stages of the ethylene glycol and PEG thermal decomposition [6,7]. The peaks of the PEG-coated sample are slightly more intense, which also indicates the presence of polyethylene glycol molecules on the surface.

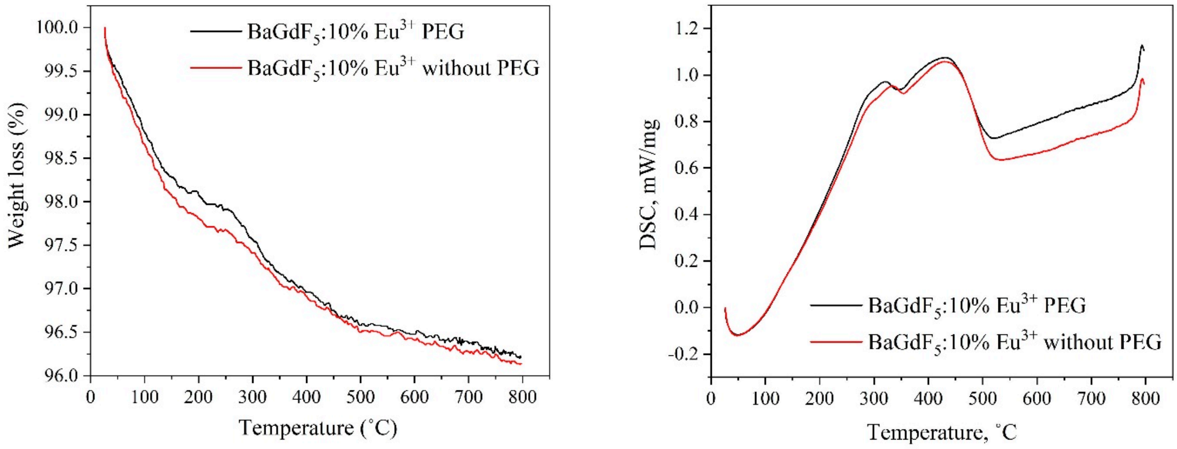

(**a**) (**b**)

Figure S2. TGA (**a**) and DSC (**b**) curves of PEG-coated and uncoated particles.

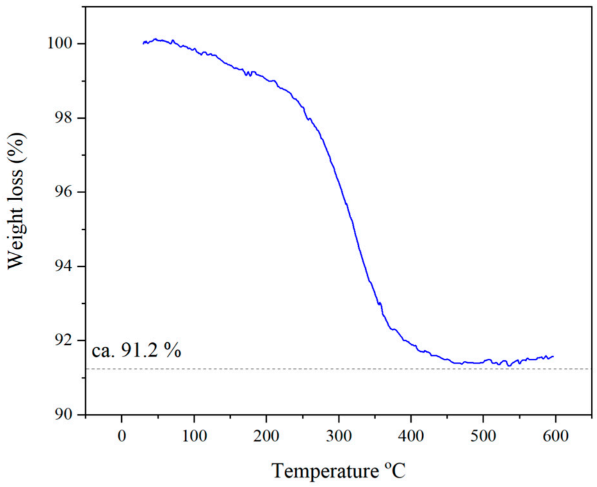

Figure S3. TGA curve obtained for $BaGdF_5$:10%Eu^{3+}@Cit^{3-} nanoparticles taken upon nitrogen atmosphere.

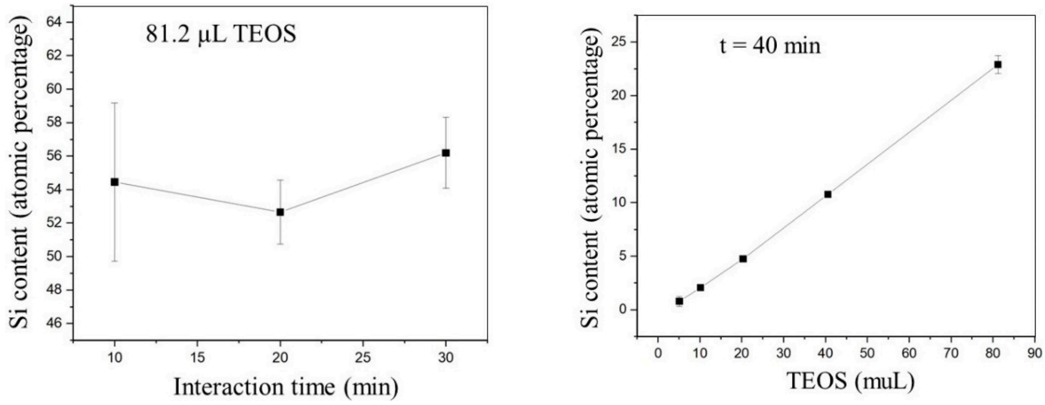

(**a**) (**b**)

Figure S4. Si at. % determined from XRF for $BaGdF_5$:10%Eu sample as obtained after two different SiO_2 method: (**a**) with fixed amount of loaded TEOS and varied interaction time and (**b**) with fixed interaction time and varied amount of TEOS.

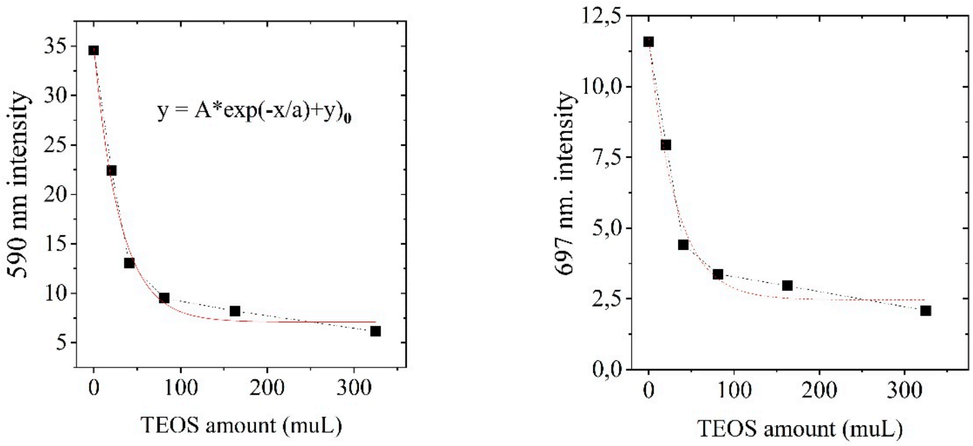

(**a**) (**b**)

Figure S5. The intensity of XEOL peaks intensity at 590 (**a**) and 697 nm (**b**) obtained for the series of $BaGdF_5$:10%Eu nanophosphors coated with different amount of SiO_2. The red curves correspond to the trend lines obtained via standard exponential decay fit.

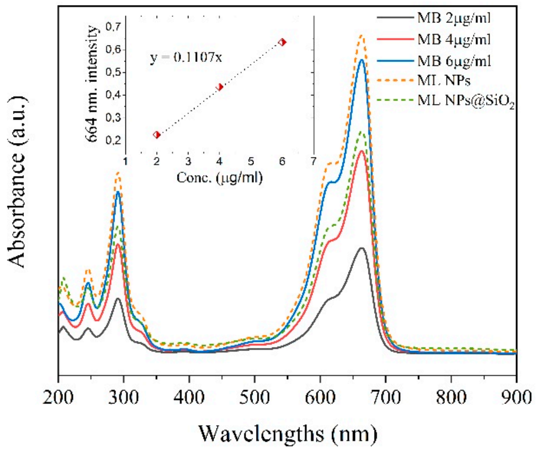

Figure S6. UV-vis absorbance spectra obtained for a series of stock MB solutions with different concentration and for mother liquors collected after nanoparticles impregnation with MB solution (8 μg/ml). Dashed orange and green curves correspond to mother liquors obtained for nanoparticles and SiO_2-coated nanoparticles impregnation, respectively. The inset demonstrates calibration curve for quantification of absorbed MB.

References

1. Banski, M.; Noculak, A.; Misiewicz, J.; Podhorodecki, A. Rice oil as a green source of capping ligands for GdF_3 nanocrystals. *New Journal of Chemistry* **2016**, *40*, 7928-7934, doi:10.1039/c6nj01052k.
2. Zhang, P.; He, Y.; Liu, J.; Feng, J.; Sun, Z.; Lei, P.; Yuan, Q.; Zhang, H. Core–shell $BaYbF_5$:Tm@$BaGdF_5$:Yb,Tm nanocrystals for in vivo trimodal UCL/CT/MR imaging. *RSC Advances* **2016**, *6*, 14283-14289, doi:10.1039/C5RA22991J.
3. Xu, C.; Ma, M.; Yang, L.; Zeng, S.; Yang, Q. Upconversion luminescence and magnetic properties of ligand-free monodisperse lanthanide doped $BaGdF_5$ nanocrystals. *Journal of Luminescence* **2011**, *131*, 2544-2549, doi:10.1016/j.jlumin.2011.06.022.
4. Chieng, B.; Ibrahim, N.; Yunus, W.; Hussein, M. Poly(lactic acid)/Poly(ethylene glycol) Polymer Nanocomposites: Effects of Graphene Nanoplatelets. *Polymers* **2013**, *6*, 93-104, doi:10.3390/polym6010093.
5. Zhang, H.; Wu, H.; Wang, J.; Yang, Y.; Wu, D.; Zhang, Y.; Zhang, Y.; Zhou, Z.; Yang, S. Graphene oxide-$BaGdF_5$ nanocomposites for multi-modal imaging and photothermal therapy. *Biomaterials* **2015**, *42*, 66-77, doi:10.1016/j.biomaterials.2014.11.055.
6. Tippabattini Jayaramudu; Gownolla Malegowd Raghavendra; Kokkarachedu Varaprasad; Gangireddygari Venkata Subba Reddy; A. Babul Reddy; K. Sudhakar; Sadiku, E.R. Preparation and characterization of poly(ethylene glycol) stabilized nano silver particles by a mechanochemical assisted ball mill process. *Journal of Applied Polymer Science* **2015**, *1*, 1-8, doi:10.1002/APP.43027.
7. Qian, T.; Li, J.; Feng, W.; Nian, H. Enhanced thermal conductivity of form-stable phase change composite with single-walled carbon nanotubes for thermal energy storage. *Sci Rep* **2017**, *7*, 44710, doi:10.1038/srep44710.
